# Emerging Advances to Transform Histopathology Using Virtual Staining

**DOI:** 10.34133/2020/9647163

**Published:** 2020-08-25

**Authors:** Yair Rivenson, Kevin de Haan, W. Dean Wallace, Aydogan Ozcan

**Affiliations:** ^1^Electrical and Computer Engineering Department, University of California, Los Angeles, CA, USA; ^2^Bioengineering Department, University of California, Los Angeles, CA, USA; ^3^California NanoSystems Institute (CNSI), University of California, Los Angeles, CA, USA; ^4^Department of Pathology and Laboratory Medicine, Keck School of Medicine of USC, Los Angeles, CA, USA; ^5^Department of Surgery, David Geffen School of Medicine, University of California, Los Angeles, CA, USA

## Abstract

In an age where digitization is widespread in clinical and preclinical workflows, pathology is still predominantly practiced by microscopic evaluation of stained tissue specimens affixed on glass slides. Over the last decade, new high throughput digital scanning microscopes have ushered in the era of digital pathology that, along with recent advances in machine vision, have opened up new possibilities for Computer-Aided-Diagnoses. Despite these advances, the high infrastructural costs related to digital pathology and the perception that the digitization process is an additional and nondirectly reimbursable step have challenged its widespread adoption. Here, we discuss how emerging virtual staining technologies and machine learning can help to disrupt the standard histopathology workflow and create new avenues for the diagnostic paradigm that will benefit patients and healthcare systems alike via digital pathology.

## 1. Introduction

The application of computational techniques to biomedical imaging is almost a century-old practice. One field that made a relatively quick transition to the digital age is radiology, where imaging modalities such as computed tomography (CT) and magnetic resonance imaging (MRI) adopted digitization and computation for storage, reconstruction, and display of medical images. However, until very recently, the field of pathology has not demonstrated similar progress toward digitization. Pathologic diagnoses are based on visual interpretations of very thin slices of tissue specimens that have been affixed to glass slides and stained. These tissue sections are the fundamental diagnostic material of pathology and cannot be as readily digitized as a radiologic film study, which has delayed the adoption of many computational techniques in pathology. Recently, automated slide scanners have been developed, which allow pathology slides to be scanned using optical microscopy and produce digital image files, referred to as whole slide images (WSIs). These WSIs bring a wide variety of potential advantages such as improving workflow, enabling telemedicine to perform rapid expert consultations from anywhere in the world, and opening up the possibility of Computer-Aided-Diagnoses (CAD) to improve patient care and speed up the diagnostic process. However, due to significant infrastructural implementation costs, along with some technical and practical limitations, the pathology industry has been slow to undergo a large-scale digital transformation. Therefore, if the digitization process can reduce operating costs, the impact on the rate of digital adoption in pathology could be substantial.

While much of the initial push toward slide digitization came from interest in research and development toward, e.g., machine learning-assisted diagnosis [1], recent advancements in imaging technologies, and image reconstruction and transformation algorithms have created new opportunities that have the potential to change the landscape of how pathology is practiced. These emerging technologies, which will be discussed in this article, aim to disrupt the histopathology process from the surgery room to the pathology lab, all the way to the telepathology diagnostic portal.

## 2. Microscopic Contrast in Tissue Imaging

Before microscopic tissue examination can take place, the sampled tissue undergoes multiple preparation and processing stages that comprise the histopathology workflow. Any examination begins with the extraction of tissue using one of the various forms of tissue sampling such as a fine needle aspiration biopsy of a discrete lesion or surgical excision of a large mass during surgery. Once the tissue is extracted, it is stained to bring contrast to various microscopic features of tissue. While there are a wide variety of different histological stains, the standard stain used by pathologists is the hematoxylin and eosin stain (H&E), and there are two main workflows used to produce it.

The primary histopathology workflow involves tissue fixation (usually in formalin, a formaldehyde-based solution), followed by processing the formalin-fixed tissue using reagents to embed it within paraffin blocks. After the paraffin embedding stage, the paraffinized tissue block is cut using a microtome to produce sections that are typically 2-4 microns thick. Once the tissue sections are cut, the paraffin blocks are carefully placed in long-term storage, per College of American Pathologists guidelines [2]. The thin sections cut from the paraffin block are hydrated and placed on a microscope slide, and then deparaffinized and finally stained to provide the coloring and contrast that is necessary for slide examination. The final step is the application of a coverslip to the slide which protects the tissue and allows for permanent storage after review by a pathologist. This embedding and staining process takes approximately 2 hours for standard H&E staining.

The second important histopathology workflow is rapid H&E fixation and staining, which is necessary to support clinical decision-making during, e.g., surgery, when the surgeon needs to understand the nature of the tissue to determine whether more sampling is needed or not. In this scenario, the pathologic evaluation requires rapid creation of the H&E slide. The process to quickly evaluate a tissue specimen for pathologic analysis is called a “frozen section” (as the tissue is frozen in a block of optimal cutting temperature compound rather than a paraffin block), and the slide staining is a swift process (freezing and staining takes approximately 10-15 minutes). However, this process is labor intensive and typically results in a lower quality stain in comparison to those generated through paraffinized tissue processing. Part of the decreased H&E stain quality is due to variability in staining by different technicians, difference in tissue size on the slides, and potential changes in the concentration of the staining reagents throughout the day.

As described above, histopathology tissue processing can be a laborious and delicate process but is a prerequisite to the process of histological staining. Most tissues demonstrate exceptionally low contrast when examined under a brightfield microscope, necessitating the staining step. By applying chemicals, different chromophores (dyes) are attached to different tissue constituents, highlighting these features to allow the microscopic examination of tissue with sufficiently rich and useful contrast.

Histological staining in various forms has been in use for the last 150 years and is still universally applied in the histopathologic assessment of every disease study in tissues. For example, the H&E stain is used as the primary stain for all cancer diagnoses. H&E results in high contrast for the nucleus, cytoplasm, and extracellular constituents, all in shades of blue (hematoxylin) or red (eosin). Other staining methods, called special stains, can provide a particular contrast for specific tissue or cellular components, which can provide an extra diagnostic dimension that is sometimes necessary to complete the pathologic evaluation process. Special stains include, e.g., periodic acid Schiff (PAS), Masson’s trichrome (MT), Jones’ methenamine silver (JMS), and reticulin silver (RS) stains, which are used routinely in the evaluation of kidney, liver and skin diseases, among many others [3]. More advanced categories of staining include, e.g., immunohistochemistry (IHC) and fluorescence in situ hybridization (FISH) that provide molecular information on antigenic and genotypic features of tissue sections [3].

As effective as the histopathology process is, it has several known drawbacks. For example, it is destructive in nature and may exhaust the tissue sample, requiring repeat tissue sampling (e.g., extra biopsies); furthermore, the tissue processing is time consuming and laborious, especially if special stains or advanced stains are involved. Therefore, in recent years, many attempts have been made to change and modernize the current histopathology workflow. The common denominator for these methods is the use of an optical instrument which generates an alternative contrast to standard histological staining. The adoption of these alternative contrast mechanisms along with novel computational methods should provide the clinicians (or computer algorithms) a result that is of similar or superior quality in comparison to the gold standard for diagnosis [4-6].

The optical imaging methods with alternative contrast that were explored over the last two decades all aimed to alter parts of the current histopathology tissue processing workflow. Many of these methods were targeted at eliminating the tissue fixation step and provide an intraoperative or bedside instrument that can be used for tumor margin assessment during surgery, in lieu of the current workflow, as what is also known as “slide-free” histopathology. Some of these methods utilized completely label-free imaging, including, e.g., multimodal nonlinear microscopy [7, 8], stimulated Raman spectroscopy [9], and photoacoustic microscopy [10]. Other methods have used a rapid staining procedure involving, e.g., acridine orange to enhance the nuclear content and differentiate it from connective tissue. Some of these methods include surface UV excitation [11], single-photon light-sheet absorption microscopy [12], and two-photon absorption microscopy [13]. In addition to these, optical coherence tomography [14, 15], reflectance confocal microscopy [16], and nonlinear optical endomicroscopy [17] have also been demonstrated for *in vivo* imaging. It is worth noting that many of these methods were demonstrated primarily for depth (volumetric) imaging [10, 12, 14, 16], while other methods have been demonstrated for surface imaging on the excised specimen [7, 9, 11, 13].

Some of these methods have also augmented their results with a postprocessing step that generates an H&E-like image. These synthetic H&E images [9, 11, 12, 18] usually require multimodal imaging (e.g., one image for the nuclear content and another for the extracellular constituents). The resulting image enables the visualization of the nuclear organization in an H&E-like fashion. This type of H&E postprocessing is important for a few reasons. For example, it can facilitate a better interpretation experience, as pathologists are typically trained to work with thin-sectioned histologically stained specimen. In addition to this, automated image segmentation and whole slide disease classification, which represent annotated [1] or weakly supervised [19] machine learning cases, have shown great promise to support pathology practitioners and these efforts have mainly focused on cancer diagnosis using H&E stained slides. However, the quality of these pseudo-H&E images usually lags behind the quality that pathologists are used to work with and has limitations in representing pigments. An additional drawback is that this type of pseudo staining is much more challenging to apply to other types of stains beyond H&E. Another limitation of the abovementioned synthetic staining methods is that they use a pixel-to-pixel mapping function (between the input and output images) [9, 12, 20], and in this sense, they ignore the microscopic texture information of an acquired input image.

Other than these methods, alternative contrast imaging has also been investigated for fixed, thin-sectioned, label-free slides [4, 5, 21]. Unlike the manually-tuned pixel-to-pixel color mappings discussed earlier, these techniques utilized data-driven learnable staining algorithms. For example, a multilayer perceptron model was used to map a spectroscopic feature vector from a single location on the tissue sample (obtained using, e.g., Fourier-transform-infrared spectroscopy [21]), into a single output vector that represents the target color of that pixel, corresponding to RGB or any other color space. This approach also ignores the microscopic texture information of the tissue as this color output is independently performed at each location of the sample, pixel-by-pixel.

As will be discussed in the next section, recent studies have also demonstrated the use of modern deep learning methods to perform texture-to-texture based image transformations to virtually stain label-free tissue sections, without a reduction in image quality.

## 3. Deep Learning-Based Virtual Staining

Modern deep learning methods typically rely upon the optimization of multilayered convolutional neural networks (CNNs) for a machine learning task [22]. Each layer of the network contains tens to hundreds of convolution kernels (filters). Each of these convolutional layers is regulated by a nonlinear activation function before transferring the information to the next layer. Deep neural networks require a one-time training effort to optimize these layers, where one of the most popular training paradigms is known as supervised learning. Supervised learning is especially the method of choice when there is access to a sufficiently large amount of training data, which contain the inputs to the network along with the matching “gold standard” ground truth information. Using this dataset, the network and its weights are then trained iteratively to minimize the difference between its prediction (i.e., the output signal, which results from a given input) and the corresponding ground truth.

Following the training process, the deep network can be used to rapidly perform its inference task in a single forward-pass, without the need for any further iterations or optimization. Both this blind inference step, as well as the training of the neural network take advantage of the availability of modern computer hardware such as graphical processing units (GPUs) and specially designed application-specific integrated circuits (ASICs) as well as tailored coding and optimization environments [23, 24]. In recent years, CNNs have proven to be very effective for implementing machine vision tasks such as image classification[25-27], annotation [28], and segmentation [29]. One of the notable advantageous properties of CNNs for handling/processing images is their shift-equivariance (or filter sharing) property, which allows them to be identically applied to arbitrary regions of interest or fields-of-view, without being limited to specific image sizes or positions.

When considering the application of deep learning-based methods to the problem of virtual staining of label-free tissue, the input to the staining network needs to demonstrate some form of meaningful contrast, presenting cues to the network for it to learn the transformation to the desired histochemical contrast. Various imaging modalities have been used to generate the needed contrast based on endogenous agents within label-free tissue to enable deep learning-based virtual staining; some examples include autofluorescence imaging [4], quantitative phase imaging [5, 30], hyperspectral imaging [31], and others [32, 33]. These deep learning-based methods successfully demonstrated virtual staining of multiple organs with H&E as well as some of the special stains (e.g., Masson’s trichrome and Jones stain) [4, 5], which provide additional channels of diagnostic information, on top of H&E.

Unlike the previously described synthetic tissue staining methods (Section 2) that perform a pixel-to-pixel transformation, CNN-based virtual staining methods learn to map/transform an input image patch to the desired target histological stain patch, by learning the relationships between the input and output data structures and distributions. The input distribution is determined by the used contrast mechanism, the microscopy technique, and the tissue type, while the output distribution is determined by the combination of the target stain and tissue type. As previously mentioned, the learning process between the input and target images involves the adjustment of the filter weights of the deep neural network to minimize a predefined cost function that compares the network output image and the desired target image. While hand-crafted cost-functions provide a good solution for many cases, they can also become data adaptive by training the network using the GAN framework [34]. In a GAN framework, two “competing” deep neural networks are trained simultaneously. The first network is known as the Generator network, which performs the desired image transformation (virtual staining), while the second network, the Discriminator (or the “Critic”), learns to discriminate between the generated images (i.e., the virtually stained images created by the Generator) and the histologically stained images that constitute the ground truth. The cost function of the Discriminator is then used as part of the Generator’s cost function. In that way, the Generator gets feedback from the Discriminator regarding the similarity of the virtual staining distribution to the histological staining distribution. This pushes the Generator to create images that will drive the Discriminator to not be able to differentiate between the generated images from the label-free specimen and those that originate from the actual histological staining process. This property of the GAN training is the source of its data-adaptive optimization.

One general criticism of GANs is that they can mislead the Generator network when being used to mimic a sought target distribution. In other words, the output microscopic image can look like it was generated from the target distribution, and therefore, it is susceptible to hallucinations [35]. To mitigate this, one approach is to constrain the cost function to be dependent not just on the Discriminator loss (that makes the image to look more like the target distribution), but also on a more standard per-pixel (i.e., structural) loss term [36]. This combined cost function regularizes the GAN training and helps to mitigate possible hallucinations at the network output. To achieve this, however, high precision registration between the input and target images is required (also known as paired images). To accurately pair the input and output images during the training phase, an extensive registration process is typically required. However, from the authors’ own experience[4, 5, 36-40], this one-time image registration effort is necessary to allow the network to learn the virtual staining-related transformation and not be affected by pixel misalignments or image registrations errors. For this goal, optical microscopy has major advantages in comparison to photography or lower resolution medical imaging techniques such as MRI or ultrasound, as it permits the user a high degree of control and nanoscopic precision over the specimen, illumination, and the microscope hardware, which have been crucial for accurate registration (or pairing) between the input and target images during the training phase of virtual staining networks. This training process, involving precise image registration protocols, is a one-time effort and does not constitute a major obstacle for wide-scale use of virtual staining technologies as it is not required in the blind inference, testing phase.

An example of the integrated image processing workflow used to prepare a training dataset for virtual staining is shown in Figure 1. The multistep image registration process begins with a global, coarse registration at the slide level, which is improved to be accurate at the local patch level over a series of steps. First, a correlation-based algorithm can be used to extract the matching region of interest from the brightfield image. Then, a multimodal registration algorithm can be used to perform a rigid registration that accounts for shifts, rotations, and scaling differences [41]. Using these roughly matched images, an initial “style transfer” network can be trained. Due to substantial mismatches in the image registration, this network will not perform an accurate virtual staining. However, this network can transform the label-free image into an intermediate image which highly resembles the histologically stained image. This image can then be used as a reference for a more accurate, pixel-level, elastic registration [38]. This elastic registration accounts for any other source of misalignments, including spherical aberrations from different objective lenses and the shrinkage/expansion of the tissue caused by the chemical staining procedure.

Figure 1Training and inference processes of deep neural network-based virtual staining. (a) Initial coarse registration steps used to match the images of the label-free tissue sections with the corresponding images of the histologically stained tissue sections. This image coregistration is performed by first extracting the region with the maximum correlation and then applying a multimodal rigid registration. (b) Procedure used to train the neural networks using a conditional GAN-based loss function, where *α* denotes a weight that balances between the per-pixel penalty and the global distribution loss. (c) Steps used to fine-tune the image coregistration and ensure that pixel-level coregistration accuracy is achieved through the use of an elastic transformation. The autofluorescence images are passed through a style-transfer network which can be used as an intermediate image to perform a correlation-based elastic coregistration. (d) Following its training, the network can virtually stain new cases that were never seen by the network before, by simply passing them through the deep neural network.

**(a):**
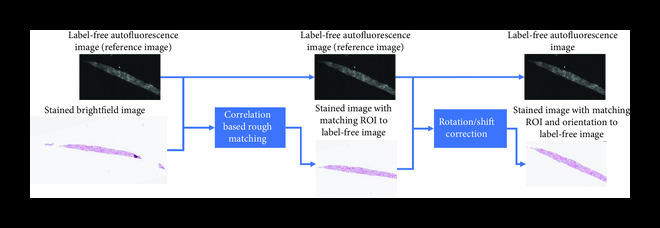
Whole slide rough registration

**(b):**
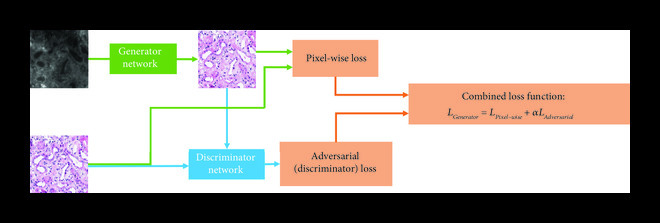
Network training

**(c):**
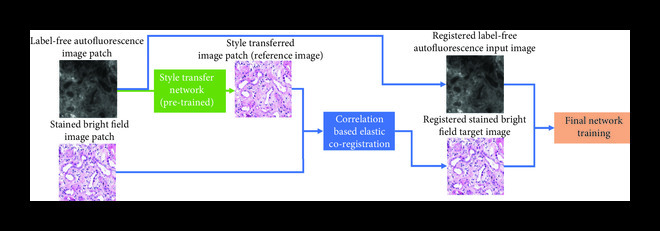
Fine training

**(d):**
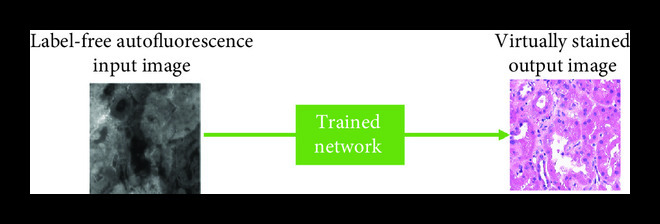
Interfere

Following this multistep registration procedure, the accurately paired input and target image patches are used for the training of the Generator CNN. The Generator will ideally be trained using a combination of pixel-based loss functions and a distribution matching loss function (Figure 1(c)). The pixel-based loss function (e.g., mean absolute error between the images) penalizes the error on the pixel level, while the distribution matching portion of the loss (i.e., the GAN loss) ensures that the images generated by the network are sharp and accurately match the distribution of the histochemically stained tissue images. At the end of the training process, which is a one-time effort, the network can be used to blindly output virtually stained tissue images of new cases that were previously not seen by the network.

**Figure 2:**
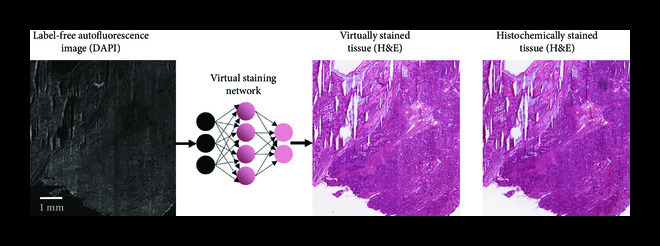
Virtual staining of a frozen section. Virtual H&E staining performed on an intraoperative consultation using rapid preparation (frozen section) of an ovarian tumor tissue section.

Once the training is complete, the quality of the virtual stains that are generated by the network must be extensively quantified and validated. While several pixel-based metrics such as mean-squared error (MSE) and structural similarity index (SSIM) can be used during the network training phase to estimate the structural loss of the network, these still cannot replace the evaluations performed by expert pathologists. Critical assessment of the virtual staining results by board-certified pathologists is currently the most comprehensive way to study the efficacy of virtual staining approaches. For example, Ref. [4] performed two types of blinded studies where expert pathologists were asked to perform diagnosis using virtually and histologically stained H&E slides and were also asked to rank/score the stain quality of histological and virtual MT and JMS stains on liver and kidney tissue sections, respectively. In this blinded study, a group of pathologists were randomly assigned to histologically stained and virtually stained images without an indicator of which one is which. The results demonstrated 100% nonmajor discordance in diagnosis between the virtually and histologically stained tissue sections. While this former study was focused on virtual staining of paraffin-fixed tissue sections, a similar performance level for virtual staining of label-free frozen tissue sections has also been demonstrated by the authors (see Figure 2), that can be used in, e.g., telepathology settings.

### 3.1. Transformative Advantages of Virtual Staining

**Figure 3:**
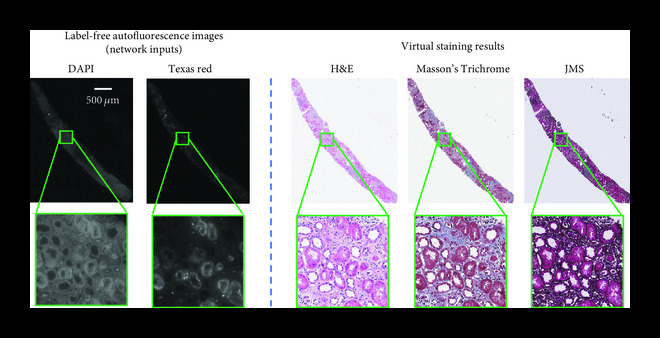
Virtual staining enables multiple stains on the same tissue section. Demonstration of multiple virtual stains (H&E, Masson’s Trichrome and Jones’ stain) applied on the same label-free kidney tissue section (needle biopsy).

Deep learning-based virtual staining opens up many exciting new avenues in digital pathology. For example, it allows for the generation of multiple virtual stains from the same physical slide section, as demonstrated in Figure 3. This capability offers numerous advantages, as the pathologists will be able to integrate information from multiple stains on the same field-of-view, which can be better used to highlight different features that are relevant for diagnosis. For example, in Figure 3, the same section (label-free tissue) is virtually stained with H&E, MT, and JMS stains, allowing the pathologist to integrate information regarding the tubulointerstitium. The H&E stain reveals tubulointerstitial inflammation, but it is suboptimal for evaluating the degree of scarring (interstitial fibrosis and tubular atrophy) and the location of the lymphocytic infiltrate. The MT stain can reveal how much interstitial fibrosis is present, and the JMS stain can reveal how much tubular atrophy is present. In contrast, unless the stain (dye) is washed away and the same sample is restained (which further adds to the labor of the histopathology workflow), the exiting tissue block will have to be cut again and additional tissue sections will need to be stained, reducing the ability to correlate features among stains, and depleting the fixed specimen. The latter can especially be a challenge in cases with tissue exhaustion as the patient might be required to undergo yet another biopsy, especially if additional tissue is needed for ancillary cancer testing.

Another exciting feature that virtual staining enables is stain blending, as well as region-of-interest or microstructure straining [6]. To exhibit these capabilities, a deep neural network that is capable of outputting multiple virtual stains was trained, where a “digital staining matrix” which represents a spatial stain map was used as part of the input to the deep network, to spatially condition the virtual stain generation. This concept is demonstrated in Figure 4(a), where a digital staining matrix on demand selects the stains to be applied for each subregion and structure of the field-of-view. Another opportunity that this framework creates is virtual stain fusion and blending as demonstrated in Figure 4(b). This technique digitally synthesizes new stains and can be used to optimize the staining process by utilizing the unique features of different stains [6].

Figure 4Virtual staining enables special stain region-of-interest highlight and stain blending. Virtual staining allows to highlight different regions of a single label-free tissue with multiple stains, simultaneously. (a) For example, the glomerular regions can be instantly stained with Jones’ stain, on top of the virtual H&E staining of the background tissue. This way, label-free tissue samples can be virtually stained with a panel of stains, following a predefined microstructure map that is decided by, e.g., a diagnostician. (b) An example of a ROI stained using H&E, Jones, and a mixture of both H&E and Jones stains (performing stain blending).

**(a):**
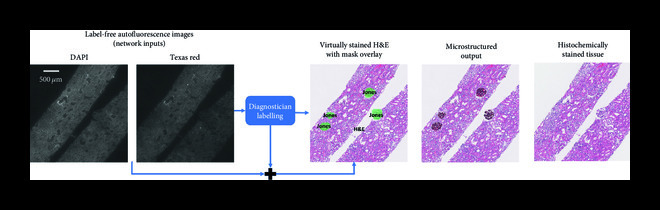

**(b):**
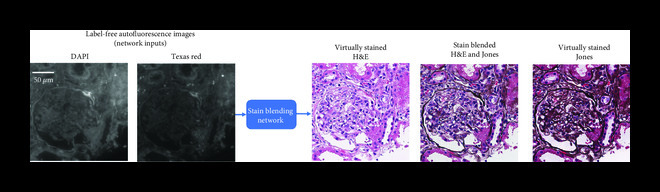

**Figure 5:**
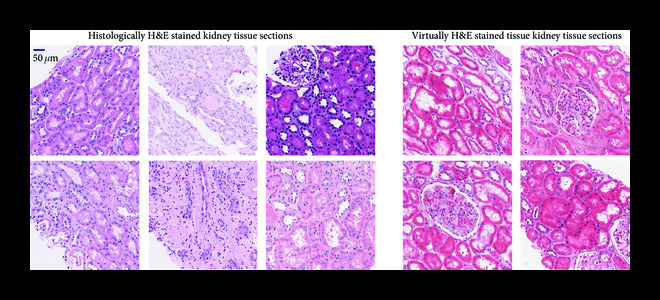
Virtual staining standardizes histological staining. Staining variability observed in histological staining performed by histotechnologists in comparison to deep learning-based virtual staining results.

An inherent property of any deep neural network that performs virtual staining is its ability to standardize the stain being generated [4]. In other words, the virtual staining network performs a uniform and consistent stain, avoiding the high level of variations commonly observed among histotechnicians or within laboratories and whole slide imaging systems [42]. This major advantage is clearly demonstrated in Figure 5, where we compare a set of kidney tissue sections stained by histotechnicians at UCLA Health against the typical fields of view that are generated by the virtual staining network, exhibiting substantially less variability from image to image. Such staining variations make the diagnostic process more challenging for clinicians as well as machine learning algorithms, as staining variability presents an important learning challenge for both humans and algorithms.

### 3.2. Stain-to-Stain Transformations

In addition to performing virtual staining of label-free tissue, there is great interest in transforming images of tissue already labeled in one manner into images labeled in another stain using deep learning. This stain-to-stain transformation capability will allow pathologists to evaluate different tissue constituents without requiring additional stains to be performed. Such an image transformation could be from a rapid stain to H&E, or from H&E to another type of stain, in which pathologists or researchers can use to perform their analysis. By transforming already existing images of histochemically stained slides, pathologists can access this additional information channel virtually, without making any changes to their existing workflow. Furthermore, many of the other benefits of previously discussed virtual staining techniques such as stain normalization also apply here for stain-to-stain transformations.

Stain transformation networks have been trained using a variety of methods, including, e.g., distribution matching losses such as CycleGANs [43]. Such distribution matching losses allow neural networks to learn how to perform transformations between imaging modalities using unmatched (or unpaired) data. Using this technique is beneficial for stain transformations due to the difficulty in creating a matched image dataset, as it can be difficult to physically/chemically convert an existing tissue from one stain to another. Several stain-to-stain transformations have been demonstrated using these techniques. For example, H&E stained tissue images have been digitally transformed into equivalent MT, fibroblast activation protein (FAP), and cytokeratin (CK) duplex stained images using CycleGANs [44].

Stain-to-stain transformations are also applicable beyond direct diagnosis. For example, by generating IHCs, they can also be used to improve the accuracy of segmentation networks [45, 46]. Similar techniques can also be used to simply normalize the color and contrast of stained tissue samples using CycleGANs [47, 48]. By performing this normalization, the consistency of the stains can be digitally improved, allowing the resulting images to be more easily used by both human diagnosticians as well as other deep neural networks, e.g., image classification.

While unpaired image datasets used by distribution matching losses are easy to generate in comparison to the matched dataset-based image preprocessing that was previously described (Figure 4), networks trained using these loss functions such as CycleGANs can be prone to hallucinations [35]. When distribution matching losses are used with unpaired image data, it can be difficult to balance the dataset and ensure that the source domain and target domain have the same distribution of features. In addition to this, it is also difficult to ensure that the transformation learned by the network is always correct.

One method used to generate a matched stain-transformation dataset and avoid the necessity of using a distribution matching loss has been to wash off the stain of the tissue sample used as the network input and to create a physically restained tissue as the matching ground truth. Using this technique, H&E stained tissue images have been used to generate virtually stained phosphohistone-H3 (PHH3) tissue images [49]. As an alternative to restaining of the same tissue section, a different network has also been trained to transform images of H&E stained tissue into MT stained images by coregistering adjacent slices, each stained with one of the two different stains [50].

## 4. Discussion and Outlook

As hospitals, doctors, and researchers look to exploit the many benefits of digital histology, the financial challenges involved with transforming histology review platforms from microscope-based to purely digital will continue to exist as a barrier to wide-scale adoption for some more time. While there are some exceptions, the majority of pathologists rely on standard microscopes to examine glass slides for their daily work. To transition every pathology practice from traditional glass slide workflows to fully digital workflows with all the necessary infrastructure, including, e.g., automated whole slide scanners, servers, storage space, data backup, and information technology (IT) support, would cost many millions of dollars. By eliminating the slide staining and reducing the slide preparation steps, virtual staining can strongly support this digital transition by introducing substantial cost savings for all laboratories and therefore make the investment worthwhile. Therefore, part of the solution to this challenge may involve using holistic analysis to understand the enterprise-wide benefits to digitizing the pathology workflow. This may reveal improvements in diagnostic turn-around-time (TAT), shorter hospital stays, and easier larger-scale histology image access for researchers and educators. This analysis will likely be institution-specific, making the accumulation of this information laborious and expensive. However, by introducing significant laboratory cost savings, virtual staining will have very easy to measure direct benefits that can offset much of the costs for digitizing the histology laboratory.

While the applications of using deep learning to perform virtual staining of label-free tissue and stain transfer have attracted much interest from pathologists, researchers and optical microscopy developers, some of its roots can be tracked to efforts over the last two decades aiming to image tissue with alternative contrast mechanisms. These initial efforts mainly focused on the applications of intraoperative slide-free imaging, which was followed by work to create H&E-like interpretations of the obtained images for easier analysis by pathologists. While most of these methods offer “frozen section” quality that can be good enough for uses such as assessment of tumor margins, they fall behind the quality and complexity that are available to modern histopathology practitioners in terms of both staining performance and variety.

The novel algorithmic approaches that are starting to bridge this gap have shown promise for histopathology applications involving thin tissue sections, and one of the next steps is to adapt them to the task of intraoperative settings, where special and advanced stains can be virtually created in almost real-time to augment the diagnostic process. By implementing a virtual staining workflow, the TAT for both paraffin processing and intraoperative frozen section processing can be greatly reduced and the image quality of the stains can be standardized. This will optimize the pathologic assessment of the tissue sections and improve the ability of deep learning algorithms to achieve Computer-Aided-Diagnoses. The transition from chemical staining to virtual staining will also reduce histology laboratory operating costs, exposure to toxic chemicals as well as sensitivity to their supply chain and quality assurance.

As discussed earlier, virtual staining technology has many advantages, such as tissue preservation by using less tissue material for analysis, which accordingly reduces the need for further biopsies by saving tissue for advanced molecular analysis. By enabling both virtual staining and molecular analysis to be performed on the exact same section of tissue, there will be a perfect correlation with advanced molecular studies. Furthermore, the ability to easily perform multiple stains on the same tissue section and region-of-interest analysis in real-time would be a game-changer for both patients and histopathologists, enabling clinicians to develop a treatment plan much quicker and shorten the time to definitive treatment.

Before this virtual staining technology can be used in clinical practice, the effectiveness of the technology must be thoroughly proven at a large scale. An appropriate clinical trial to compare the quality of the virtually stained tissue images, including the lack of diagnoses inferiority in comparison to current technology, will be required. This study should demonstrate the technology on a multitude of samples to be tested across several different independent medical centers. Furthermore, to ensure widespread adoption, the technology must be scaled to a point where it can be effectively used at a throughput required by large healthcare systems and to be easily used by technicians with limited training.

The emerging developments discussed in this paper illuminate new avenues for pathology and incentivize the adoption of digital pathology by changing the perception of digital pathology as merely another laborious step in the histopathology process into a significant workflow improvement with laboratory cost savings. Therefore, we believe that the implementation of virtual staining will present a paradigm shift for how surgical tissue samples are processed and eventually analyzed.

## 5. Materials and Methods

Human samples were obtained from the Translational Pathology Core Laboratory and were prepared by the Histology Laboratory at UCLA under IRB 18-001029 (UCLA). All of the tissue sections were obtained after the deidentification of patient-related information and were prepared from existing (i.e., archived) specimens. Therefore, this work did not interfere with standard practices of care or sample-collection procedures; the original tissue specimens were archived before this work and were not collected specifically for this research.
